# Pregnancy-associated cardiac dysfunction and the regulatory role of microRNAs

**DOI:** 10.1186/s13293-020-00292-w

**Published:** 2020-04-06

**Authors:** Laila Aryan, Lejla Medzikovic, Soban Umar, Mansoureh Eghbali

**Affiliations:** Department of Anesthesiology, Division of Molecular Medicine, David Geffen School of Medicine at University of California, Los Angeles, BH-550 CHS, Los Angeles, CA 90095-7115 USA

**Keywords:** Pregnancy, MicroRNA, Gestational diabetes, Peripartum cardiomyopathy, Pre-eclampsia, Gestational hypertension, Heart failure, Cardiac dysfunction

## Abstract

Many crucial cardiovascular adaptations occur in the body during pregnancy to ensure successful gestation. Maladaptation of the cardiovascular system during pregnancy can lead to complications that promote cardiac dysfunction and may lead to heart failure (HF). About 12% of pregnancy-related deaths in the USA have been attributed to HF and the detrimental effects of cardiovascular complications on the heart can be long-lasting, pre-disposing the mother to HF later in life. Indeed, cardiovascular complications such as gestational diabetes mellitus, preeclampsia, gestational hypertension, and peripartum cardiomyopathy have been shown to induce cardiac metabolic dysfunction, oxidative stress, fibrosis, apoptosis, and diastolic and systolic dysfunction in the hearts of pregnant women, all of which are hallmarks of HF. The exact etiology and cardiac pathophysiology of pregnancy-related complications is not yet fully deciphered. Furthermore, diagnosis of cardiac dysfunction in pregnancy is often made only after clinical symptoms are already present, thus necessitating the need for novel diagnostic and prognostic biomarkers. Mounting data demonstrates an altered expression of maternal circulating miRNAs during pregnancy affected by cardiovascular complications. Throughout the past decade, miRNAs have become of growing interest as modulators and biomarkers of pathophysiology, diagnosis, and prognosis in cardiac dysfunction. While the association between pregnancy-related cardiovascular complications and cardiac dysfunction or HF is becoming increasingly evident, the roles of miRNA-mediated regulation herein remain poorly understood. Therefore, this review will summarize current reports on pregnancy-related cardiovascular complications that may lead to cardiac dysfunction and HF during and after pregnancy in previously healthy women, with a focus on the pathophysiological role of miRNAs.

## Introduction

During pregnancy, various crucial adaptations in the cardiovascular system occur which are necessary for the progression of successful gestation [1]. Maladaptation of the cardiovascular system during pregnancy in previously healthy women can lead to complications that may cause maternal and fetal mortality [2, 3]. Cardiovascular complications during pregnancy may put the mother at risk to develop cardiac dysfunction and subsequent heart failure (HF) [2, 4]. These complications include metabolic changes such as gestational diabetes mellitus (GDM), hypertensive disorders such as preeclampsia (PE) and gestational hypertension (GH), and cardiac structural changes such as peripartum cardiomyopathy (PPCM) [5–8]. Cardiac complications in pregnancy are becoming increasingly common [9]. In the USA, about 12% of pregnancy-related deaths have been attributed to cardiac dysfunction, and having cardiac dysfunction during pregnancy has been associated with a 7.7-fold increase in the risk of death [9, 10]. Furthermore, the adverse effects of cardiovascular complications on the heart can be long-lasting, pre-disposing the mother to HF later in life [11, 12].

The heart undergoes several structural, metabolic, and functional changes during pregnancy to accommodate the enhanced cardiac output necessary for meeting maternal and fetal demands [13]. These changes are distinct from adverse cardiac remodeling which precedes HF [14]. However, GDM, PE, GH, and PPCM have all been shown to induce cardiac metabolic dysfunction, oxidative stress, fibrosis, apoptosis, and diastolic and systolic dysfunction in the hearts of pregnant women, all of which are hallmarks of HF [14]. The underlying molecular cardiac pathophysiology of these complications is not yet fully elucidated and warrants further investigation. Furthermore, diagnosis of cardiac dysfunction and HF in pregnancy is often made only after clinical symptoms are already present, thus necessitating the need for novel diagnostic and prognostic biomarkers.

MicroRNAs (miRNAs) are small, non-coding RNAs that regulate gene expression at the post-transcriptional level by binding to the 3’ untranslated region (3′ UTR) of the target mRNA, marking it for early degradation or blocking its translation [15]. MiRNAs are highly conserved between different species and may control multiple signaling pathways at once [16]. Mounting data demonstrates altered circulating miRNA expression in pregnancy affected by cardiovascular complications [17, 18]. Throughout the past two decades, circulating and tissue-specific miRNAs have become of growing interest as modulators and biomarkers of pathophysiology, diagnosis, and prognosis in a variety of cardiovascular disorders including HF [19, 20]. Although a significant number of studies have been published on the association between GDM, PE, GH, PPCM, and cardiac dysfunction or HF, miRNA-mediated regulation herein remains poorly understood.

This review will discuss current reports on pregnancy-related cardiovascular complications that may lead to cardiac dysfunction and HF during and after pregnancy in previously healthy women, with a focus on the pathophysiological role of miRNAs.

## Physiological cardiovascular changes during pregnancy

### Hemodynamics of the maternal cardiovascular system during pregnancy

The maternal cardiovascular system undergoes several changes during pregnancy. Blood flow increases to meet the metabolic needs of the maternal organs and fetus [13]. Blood volume increases approximately 45% above pre-pregnancy levels [1]. Stroke volume, heart rate, and end-diastolic volume all increase, resulting in enhanced cardiac output [1]. Indeed, cardiac output rises up to 50% above pre-pregnancy levels at about 16–20 weeks of gestation [21]. Both systolic and diastolic arterial blood pressure decrease in the first and second trimesters [21, 22]. However, arterial blood pressure rises in the third trimester, returning to baseline by the end of pregnancy [22]. To meet these hemodynamic changes during pregnancy, the heart undergoes structural and functional changes.

### Structural and metabolic changes in the heart during pregnancy

Natural volume overload, mechanical stretch, and hormonal changes during pregnancy induce physiological cardiac hypertrophy [23–25]. In contrast to pathological cardiac hypertrophy, pregnancy-induced physiological cardiac hypertrophy is characterized by proportional increases in cardiomyocyte size and therefore growth in left ventricular (LV) wall thickness and chamber dimensions [24]. Importantly, myocardial capillary density remains normal. Furthermore, pregnancy-induced physiological hypertrophy is not associated with fibrosis, cardiomyocyte sarcomere disarray, or enhanced re-expression of the cardiac fetal gene program [24]. Notably, the changes in cardiac structure and function during normal healthy pregnancy are rapidly reversed post-partum [26].

Metabolic changes in the heart during pregnancy are in contrast to those in pathological cardiac hypertrophy and HF. HF is characterized by a switch from myocardial fatty acid oxidation as a main source of energy to enhanced utilization of glucose [27]. Animal models in various studies have demonstrated that pregnancy is associated with a decrease in cardiac glucose utilization and increased utilization of fatty acids [28–30]. However, a decrease in cardiac fatty acid oxidation genes has also been reported [31]. Interestingly, cardiac insulin signaling and mitochondrial function remain unaltered in pregnancy-induced hypertrophy in mice, while they are depressed in pathological cardiac hypertrophy and HF [28, 32, 33].

### Signaling pathways regulating the cardiac phenotype during pregnancy

Cardiac molecular signaling pathways activated in pregnancy-induced hypertrophy are distinct from those activated during pathological hypertrophy [23]. Some of these pathways have been demonstrated to be regulated by miRNAs. The best characterized miRNA-regulated pathways in pregnancy-induced cardiac hypertrophy include phosphoinositide-3-kinase/protein kinase B/glycogen synthase kinase 3β (PI3K/Akt/ GSK3β) signaling, mitogen-activated protein kinase (MAPK) signaling, calcineurin signaling, and signal transducer and activator of transcription 3 (STAT3) signaling [34, 35].

#### Phosphoinositide 3-kinase (PI3K), protein kinase B (Akt), and glycogen synthase kinase 3 beta (GSK3β)

The PI3K/Akt pathway has been demonstrated as an important mediator in pregnancy-induced cardiac hypertrophy in several studies. The major target of PI3K/Akt signaling is GSK3β, an inhibitor of pathological cardiac hypertrophic signaling that becomes inactivated by Akt-mediated phosphorylation [36]. A large number of studies suggest that PI3K/Akt/GSK3β signaling is cardio-protective and mediates physiological rather than pathological cardiac hypertrophy. All three components of the signaling cascade have been shown to be of great importance for cardio-protection. Indeed, mice with cardiomyocyte-specific expression of constitutively active forms of PI3K and Akt respectively have been shown to develop cardiac hypertrophy with preserved contractility and systolic function, without cell death or fibrosis [37–41]. Furthermore, male mice with cardiomyocyte-specific expression of dominant-negative forms of PI3K and Akt respectively have a diminished physiological hypertrophic response, but enhanced hypertrophy and cardiac dysfunction in response to pressure overload by transverse aortic constriction (TAC) [38, 39]. Akt activation, as measured by phosphorylation status, is upregulated in the LV of pregnant mice and rats, during mid- and late pregnancy [34, 42]. In contrast, one study has also reported the downregulation of phosphorylated Akt in the hearts of pregnant rats compared to non-pregnant rats, which is restored postpartum [43]. This discrepancy could be explained by differences in the estrus cycle of non-pregnant control animals since estrogen levels vary during the estrus cycle in non-pregnant mice [44]. Estrogen is also known to activate MAPK and PI3K/Akt pathways [45, 46]. We have shown previously that estrogen increases tyrosine kinase c-Src activity (phosphorylation) in the heart mimicking increased c-SRC activity in the late pregnant heart [47]. In addition to GSK3β, other targets of PI3K/Akt signaling, such as the mammalian target of rapamycin (mTOR) and ribosomal S6 protein kinase (p706SK) have also been demonstrated to be upregulated in mouse hearts in mid-pregnancy [34]. Interestingly, compared to wild type (WT), mice expressing constitutively-active Akt had larger hearts when non-pregnant which did not undergo further hypertrophy [34]. Along the same lines, mice expressing constitutively active, inhibiting, GSK3β were blocked in their hypertrophic response to pregnancy [34]. Taken together, both Akt and GSK3β are important mediators of pregnancy-induced cardiac hypertrophy [34].

#### Mitogen-activated protein kinases (MAPKs)

During pregnancy, hormonal changes and mechanical stretch of cardiomyocytes alter the activation of several MAPK signaling pathways [34, 48]. MAPKs mediate various cellular responses in the healthy and diseased heart including hypertrophy, apoptosis, proliferation, differentiation, survival, and inflammatory responses [49]. In the heart, extracellular signal-regulated kinase (ERK) is protective against adverse remodeling, while p38 MAPK and c-Jun N-terminal kinase (JNK) are associated with stress responses [24, 34, 49]. Additionally, crosstalk between ERK and p38 and JNK MAPKs regulates various processes in the heart [49]. Various transgenic mouse models illustrate the importance of MAPK in physiological cardiac hypertrophy. Mice expressing cardiac-specific constitutively active MAPK kinase 1 (MEK1), a direct upstream activator of ERK1/2, but that does not activate JNK and p38, exhibit cardiac hypertrophy with enhanced cardiac function without decompensation over time, reminiscent of physiological cardiac hypertrophy [50]. However, mice lacking the p38 upstream regulator apoptosis signal-regulating kinase 1 were shown to exhibit less adverse cardiac remodeling upon pressure overload by TAC, but more pronounced physiological hypertrophy compared to WT mice [51]. ERK phosphorylation, and thus activation, is shown to be increased in LV of early pregnant rats and mid-pregnant mice [29, 34, 42]. In contrast, phosphorylation of JNK and p38 MAPK are decreased in the hearts of pregnant rats and mice [34, 43]. Furthermore, in pregnant rats, cardiac p-p38 and p-JNK levels were shown to be negatively associated with lower LV mass/volume ratio [43].

#### Calcineurin

Calcium-dependent phosphatase calcineurin is well-known to be upregulated in human hypertrophic and failing hearts and acts as a mediator of adverse cardiac remodeling by mediating nuclear translocation of the pro-hypertrophic transcription factor nuclear factor of activated T-cells (NFAT) [52, 53]. Elevated cardiac calcineurin expression and activity have been demonstrated in early pregnancy, which is partially induced by hormonal changes [54]. Blocking calcineurin using cyclosporine A diminishes the development of pregnancy-induced physiological cardiac hypertrophy in mice [54]. Interestingly, calcineurin inhibition also blocks pregnancy-induced cardiac ERK1/2 and activation [54]. While calcineurin levels remain elevated in pathological hypertrophy and HF, by late pregnancy cardiac calcineurin levels decrease dramatically [31, 54].

#### Signal transducer and activator of transcription 3 (STAT3)

STAT3 is an important cardio-protective signaling molecule and the transcription factor involved in the pathophysiology of various cardiac diseases [55, 56]. As a transcription factor, STAT3 activates several anti-apoptotic, anti-oxidative, and pro-angiogenic genes in the heart [55]. Interestingly, STAT3 has been shown to both activate and inhibit fibrotic and inflammatory genes in the heart, most likely due to differences in post-translational modifications, and cellular localization [55, 57–60]. Furthermore, STAT3 has been shown to alter miRNA expression in both the male and female hearts [61, 62]. The non-genomic actions of STAT3 include, among others, a protective function in mitochondria by regulating reactive oxygen species (ROS) production [56, 63]. In mouse heart during pregnancy and postpartum, STAT3 activation, as determined by phosphorylation status, has been shown to be protective in a number of pregnancy-related cardiac insults [64–67].

## Cardiac pathophysiology of cardiovascular complications during pregnancy

Cardiovascular complications reflect an inability to adapt to the various changes in systemic physiology that are associated with pregnancy [3]. While cardiovascular complications in pregnancy may affect multiple organ systems including the liver, kidneys, and brains [68, 69], we focus on the adverse effects on the heart. Indeed, metabolic changes in GDM, elevated blood pressure, and vascular resistance in PE and GH, and LV structural and functional changes in PPCM may all negatively affect cardiac function and may promote HF development [5–8].

### Gestational diabetes mellitus (GDM)

Maintaining glucose homeostasis is of utmost importance during pregnancy for maternal and fetal health as it ensures sufficient glucose levels to promote fetal development while simultaneously maintaining maternal nutrition [69]. GDM is characterized by de novo hyperglycemia occurring in the second or third trimester despite having no previous history of diabetes mellitus [69]. The prevalence of GDM is increasing in parallel with the rise of maternal age and obesity, and is reported to affect approximately 5–14% of pregnancies in the USA [70].

Impaired glucose homeostasis is common in patients with HF even in the absence of hyperglycemia and is likely to contribute to disease progression [71]. As such, GDM was found to be independently associated with greater LV mass, impaired LV relaxation, and LV systolic function [5]. However, GDM patients have also been shown to display only LV diastolic filling impairment without changes in LV mass or systolic function [72]. Strikingly, a history of GDM is associated with a ~ 2-fold increased risk of developing HF up to 25 years postpartum [73–75].

Several factors contribute to the pathophysiology of GDM, including insulin resistance, pancreatic β-cell dysfunction, and elevated hepatic gluconeogenesis. Insulin resistance results in impaired plasma membrane translocation of glucose transporter 4 (GLUT4), the primary transporter that is responsible for shuttling glucose into the cell as an energy source [76]. While insulin resistance decreases during normal pregnancy, insulin-stimulated glucose uptake is reported to drop by an extra 54% in GDM patients compared with normal pregnant controls, leading to hyperglycemia [76, 77]. It is important to note that there is a strong association between body weight and insulin resistance in pregnancy [78]. Women weighing more than 95 kg between 24 and 32 weeks of gestation were reported to have significantly higher levels of severe insulin resistance and in turn, a higher risk of GDM [78]. Indeed, in GDM patients, downstream regulators of insulin, including PI3K and GLUT4, have all been shown to be alternatively expressed or activated compared to healthy controls [77]. An increase in serine phosphorylation of insulin receptor substrate has been demonstrated in weeks 30 through 34 of gestation. This leads to a decrease in insulin receptor substrate association with insulin receptor and can inhibit PI3K activity, which in turn, inhibits insulin signaling from activating GLUT4 translocation [79]. Adaptation of insulin-producing pancreatic β cells is critical for a proper response to pregnancy-related insulin resistance and includes increased β cell number, size, and insulin secretion [80]. The adaptation of β cells is thought to be mediated by maternal and placental hormones including prolactin [80]. Prolactin signals through the Akt/mTOR pathway to reduce β cell apoptosis and enhance glucose-stimulated insulin secretion, and through the ERK/MAPK pathway to enhance β cell proliferation [80]. In late gestation, where insulin resistance is at its peak, the maternal system shifts towards a pro-inflammatory immune state [81], which can have adverse outcomes as β cells can be susceptible to macrophage infiltration [82]. However, the mechanism responsible for the inability of β cells to compensate in GDM is yet unknown [80]. During pregnancy, hepatic gluconeogenesis rates increase in healthy women and GDM patients [83, 84]. Together with impaired insulin secretion and sensitivity, higher levels of hepatic gluconeogenesis result in the hyperglycemia observed in GDM patients [69].

Limited research has been conducted on the molecular cardiac pathophysiology of GDM. Recently, GDM was induced in pregnant mice by intraperitoneal injection of streptozotocin (STZ) [85]. Here, retinoic acid treatment attenuated STZ-induced cardiac hypertrophy and fibrosis by enhancing expression of mitochondrial superoxide dismutase (mnSOD), decreasing oxidative stress and reactive oxygen species (ROS) levels, and dampening NF-κB signaling [85]. Changes in LV structure and function reported in GDM are similar to those in diabetic cardiomyopathy [86]. As such, it is tempting to hypothesize that GDM cardiac pathophysiology includes dysregulated insulin/PI3k/Akt/mTOR-mediated autophagy, MAPK-mediated inflammation, mitochondrial dysfunction, apoptosis, and cardiac microvascular dysfunction as is observed in diabetic cardiomyopathy [86].

### Preeclampsia (PE) and gestational hypertension (GH)

In the USA, up to 10% of all pregnancies are complicated by hypertensive disorders [87]. Ranging in severity, hypertensive pregnancy disorders can be classified as preeclampsia-eclampsia, gestational hypertension, pre-existing chronic hypertension, and PE superimposed on pre-existing chronic hypertension [88]. Here, we will focus on de novo-developed PE and GH.

#### Preeclampsia (PE)

PE complicates 5 to 7% of pregnancies and remains the main cause of maternal and fetal morbidity and mortality [89]. Up to now, the only definitive treatment for PE is delivery of the fetus and placenta; however, in some cases, PE can persist or develop postpartum [68]. Currently, PE is diagnosed based on de novo hypertension after 20 weeks of gestation with a systolic BP of ≥ 140 mm Hg or diastolic BP ≥ 90 mm Hg, and in severe cases ≥ 160 mm /≥ 110 mm Hg [68]. Furthermore, at least one other symptom indicating maternal organ dysfunction including kidney, liver, neurological and hematological complications, will be present [68, 87].

Elevated systemic vascular resistance in PE may adversely affect cardiac structure and function and as such, PE is associated with both short- and long-term cardiovascular events, including adverse cardiac remodeling and HF [6]. In various stages of disease progression, PE patients have been reported to exhibit decreased cardiac output, higher LV afterload, increased LV mass and LV wall thickness and LV diastolic dysfunction [90–98]. Strikingly, women with previous early-onset of preeclampsia have significantly higher fasting blood glucose, insulin, triglycerides, and total cholesterol levels as compared to women with late-onset preeclampsia at the time of follow-up even 3 months postpartum [99]. The increase in these risk factors indicates a higher risk of future CVD in women with previous early-onset preeclampsia [99]. These results highlight the significance of early prevention for patients with preeclampsia.

The exact etiology of PE is still controversial, but placental ischemia seems to play a central role in its onset [68]. The later phase in PE pathophysiology is characterized by elevated circulating levels of the anti-angiogenic factors, a pro-inflammatory state and alterations in the renin-angiotensin pathway and sympathetic nervous system (SNS) [68]. The anti-angiogenic soluble fms-like tyrosine kinase-1 (sFLT1) exerts its effects by binding to the pro-angiogenic protein vascular endothelial growth factor (VEGF) and placental growth factor (PIGF), thus inhibiting their biological activity and causing systemic endothelial dysfunction [100, 101]. Soluble endoglin (sENG) is a transforming growth factor-β1 (TGF-β1) inhibitor and may potentiate sFLT1 vascular effects [102]. Reduced levels of anti-inflammatory cytokine IL-10 and elevated complement system signaling in PE patients contribute to a pro-inflammatory state in PE [103, 104]. Enhanced sensitivity to angiotensin II has been reported in PE patients, despite reduced circulating renin and angiotensin II levels [105]. Furthermore, PE patients are reported to exhibit elevated sympathetic nerve activity [106]. Together, these changes lead to a high systemic vascular resistance state and hypertension in the mother [68, 107].

Novel players have recently emerged in the cardiac pathophysiology of PE. Mutations in the atrial natriuretic peptide-converting enzyme, also known as corin, and transcription factor storkhead box 1 (STOX1) have been shown to associate with PE [108, 109]. Recent studies using transgenic mouse models of corin and STOX1 have demonstrated their role in PE-induced cardiac pathology [110, 111]. Corin-deficient mice or mice expressing mutated corin developed cardiac hypertrophy during pregnancy which persisted postpartum [110]. Pregnant mice with feto-placental STOX1 overexpression developed cardiac hypertrophy with enhanced fibrosis, together with the upregulation of genes involved in renin-angiotensin signaling [111].

#### Gestational hypertension (GH)

GH is a form of hypertension that appears de novo after 20 weeks of gestation, but in contrast to PE, does not involve dysfunction of other organ systems [87]. GH affects 6 to 7% of pregnancies and is diagnosed as systolic BP of ≥ 140 mm Hg or diastolic BP ≥ 90 mm Hg without proteinuria [87, 89]. While GH is a risk factor for PE, it is important to note that GH and PE are separate disorders. It is yet unclear whether GH etiology is distinct from PE. However, the inflammatory response signature is shown to be different between patients with GH and PE [112].

Cardiac LV structure and function in GH patients is altered compared to normotensive pregnant women. Patients suffering from GH have been reported to exhibit reduced ejection fraction (EF), alterations in end-systolic volume, increased LV mass and wall thickness, and LV diastolic dysfunction in varying degrees [7, 95, 113–117]. However, cardiac impairments in GH patients are not as large as in PE patients, likely because PE is not encompassed by hypertension alone, but rather a multi-organ system disorder [7]. Like with PE, women with a history of GH remain at an increased risk of developing HF later in life [118].

### Peripartum cardiomyopathy (PPCM)

PPCM is a rare but life-threatening pregnancy-related cardiac disease which presents itself with HF secondary to LV dysfunction, either towards the end of pregnancy or within five months postpartum [119, 120]. The incidence of PPCM is approximately 1 in 1000–4000 live births in the USA and is diagnosed as an EF < 45% [8, 120]. While women often recover to normal cardiac function, long-lasting morbidity and mortality are present in up to 77% of PPCM patients [8, 121–124]. The exact etiology of PPCM is yet unknown; however, hormonal and vascular changes, as well as genetics seem to play a role [8]. Key features of PPCM pathophysiology include oxidative stress, endothelial dysfunction, angiogenic imbalance, and inflammatory reactions [125].

The anti-angiogenic 16-kDa N-terminal fragment of the nursing hormone prolactin (16 kDa-PRL) has been identified as a potential driving factor of PPCM [64]. Prolactin may be cleaved by cathepsin D [64]. Elevated serum levels of cathepsin D were found in PPCM patients and PPCM mouse models [64, 126]. Accordingly, 16 kDa-PRL levels are upregulated in the serum of PPCM patients and suppression of PRL secretion from the pituitary with the dopamine D2 receptor agonist bromocriptine had a beneficial effect in clinical trials on PPCM outcome [64, 127, 128]. How 16-kDa-PRL causes vascular dysfunction remains unclear, but is thought to involve inhibition of pro-angiogenic mediator plasminogen activator-1 (PAI-1) and regulation of miRNA expression [62, 129]. Enhanced 16-kDa-PRL levels in PPCM are thought to be caused by impaired activation of STAT3. Cardiomyocyte-specific STAT3-deficient mice develop PPCM [64]. Cardiac cathepsin D expression is elevated in these female STAT3-deficient mice, which is associated with enhanced production of 16-kDa-PRL. It was demonstrated that STAT3 deficiency led to diminished levels of mnSOD in cardiomyocytes, leading to increased oxidative stress that promotes the release of cathepsin D [64]. As a result, cardiomyocyte-specific STAT3-deficient female mice exhibited enhanced cardiac fibrosis, endothelial cell death, decreased cardiac capillary density and systolic dysfunction [64]. Importantly, decreased myocardial STAT3 expression was found concomitant with elevated serum cathepsin D and 16 kDa-PRL in PPCM patients [64].

Another factor participating in PPCM pathophysiology is the imbalance of pro-angiogenic VEGF and anti-angiogenic sFlt1 [125, 130]. The peroxisome proliferator-activated receptor-gamma coactivator 1-alpha (PGC-1α) is a transcriptional regulator of metabolic and angiogenic pathways in numerous tissues, including the heart [131]. Similar to STAT3 cardiac knockout mice, mice lacking PGC-1α in cardiomyocytes develop PPCM [130]. PGC-1α-deficient female mice exhibit decreased secretion of VEGF from cardiomyocytes, thus dramatically lowering the threshold for cardiac sFLT1 toxicity. Stimulation of sFLT1 caused enhanced systolic dysfunction in cardiomyocyte PGC-1α-deficient mice, while only affecting diastolic dysfunction in WT mice [130]. Importantly, plasma levels of sFLT were enhanced in PPCM patients compared to healthy pregnant women [130]. Additionally, part of PPCM pathophysiology is attributable to PGC-1α-deficiency causing mnSOD downregulation and thus elevated oxidative stress and cardiac capillary dysfunction [130].

Inflammation has also been proposed as a possible underlying mechanism of PPCM pathophysiology [125]. Elevated plasma levels of pro-inflammatory cytokines such as c-reactive protein (CRP), interleukin-6 (Il-6), tumor necrosis factor-α (TNF-α), and interferon-γ (IFN-γ) have been found in PPCM patients and were shown to positively correlate with cardiac dysfunction [121, 126, 132].

Finally, a recent genetic study has identified 26 distinct truncating variants in eight genes in PPCM patients as compared to the reference population [133]. The majority of the identified truncating variants were in the titin gene and were observed in 10% of PPCM patients compared to ~ 1% in the reference population [133]. The sarcomeric protein, titin, contributes to homeostasis of sarcomere structure and is essential for coordinated cardiomyocyte contraction [134]. Interestingly, deleterious titin mutations have also been found in similar proportions in patients with idiopathic dilated cardiomyopathy [133].

## Cardiac-related miRNAs in pregnancy-related cardiovascular complications

While up to 75% of the genome is transcribed into RNA, only 2% of the genome consists of protein-coding genes [15]. Consequently, non-coding RNAs, and in particular small non-coding miRNAs, have emerged as critical regulators of cellular processes in both health and disease [15]. In turn, many miRNAs are dynamically regulated by disease states. Indeed, numerous studies have shown changes in miRNA profiles during pregnancy with complications [17, 18]. Various cell types actively secrete miRNAs into the circulation, and thus can both mediate crosstalk between different cell-types or organs, and simultaneously represent disease biomarkers [135]. It has been shown that many miRNAs that are differentially expressed in maternal serum or plasma originate from the placenta [136, 137]. Since miRNAs are well-known to mediate various crucial processes in HF development [19], it is appealing to hypothesize that at least part of the cardiac dysfunction and HF pathophysiology in pregnancy-related complications may be mediated by miRNAs.

### Dysregulated miRNAs in gestational diabetes mellitus

**Fig. 1:**
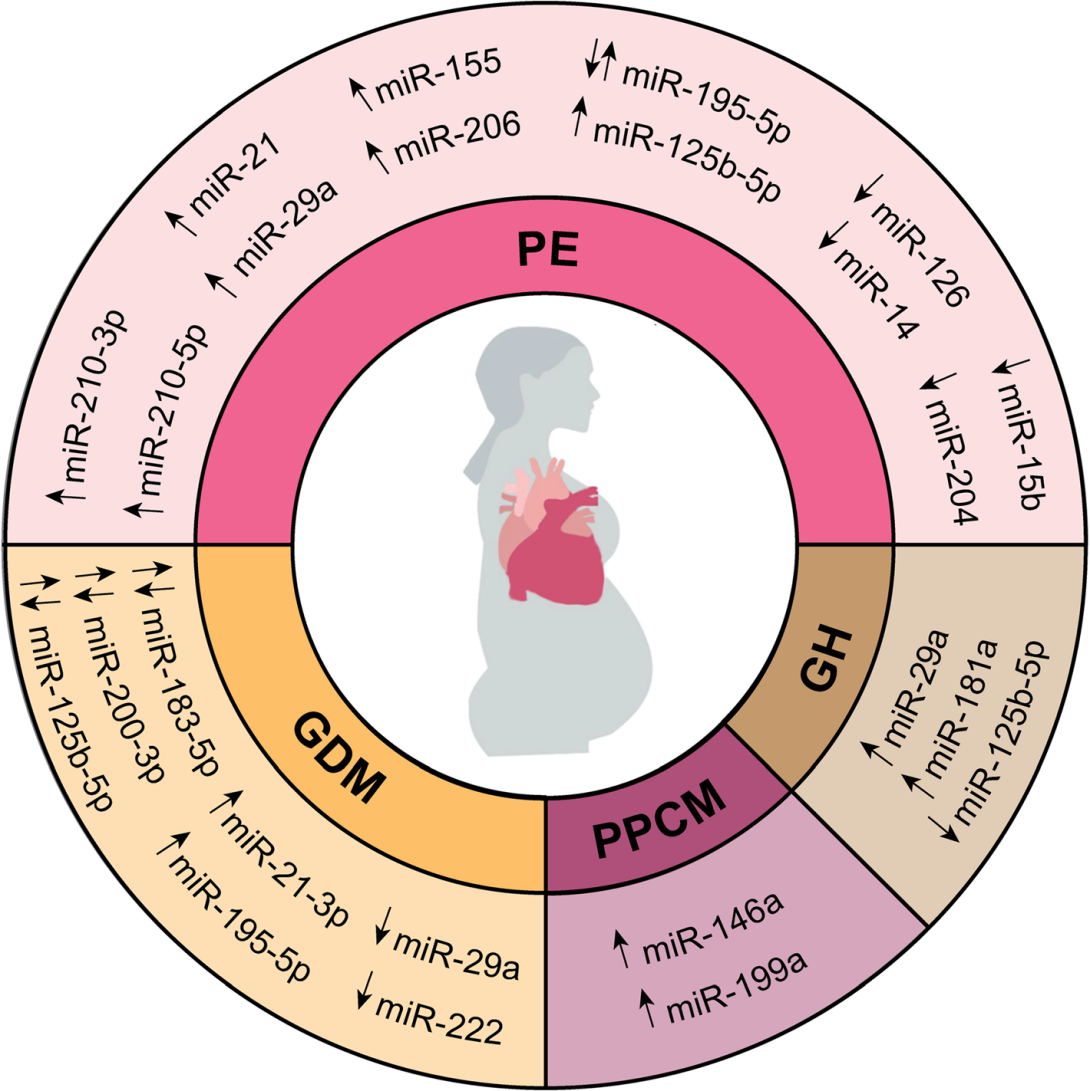
Dysregulated circulating cardiac-related miRNAs in cardiovascular complications during pregnancy. Depicted miRNAs have been shown to be involved in animal models of heart disease. GDM, gestational diabetes mellitus; GH, gestational hypertension; miR, microRNA; PE, preeclampsia; PPCM, peripartum cardiomyopathy

Several circulating miRNAs have been shown to be expressed differentially in patients with GDM. Here, we will discuss those miRNAs which have already been implicated in the pathophysiology of diabetic cardiomyopathy or other forms of adverse cardiac remodeling and HF (Fig. 1 and Table 1).

**Table 1:** Differentially expressed circulating miRNAs in gestational diabetes mellitus and their effects in heart disease

miRNA	Regulation in human pregnancy	Ref.	Heart disease	Mechanism and outcome	Ref.
miR-125b-5p	↑ serum in first trimester, normalizes in second and third	[138]	Mouse LAD ligation	↓ bak1, ↓ klf13 → ↓ cardiomyocyte apoptosis	[139]
miR-183-5p	↑ serum, first trimester ↓ serum, third trimester	[138]	Rat cardiac IRI	↓ VDAC1 → ↓ apoptosis	[140]
miR-200b-3p	↑ serum, first trimester ↓ serum, third trimester	[138]	Mouse STZ-induced diabetic cardiomyopathy	↓ CD36, ↓ EndoMT → ↓ cardiac fibrosis ↓ cardiac dysfunction	[141, 142]
miR-21-3p	↑ plasma	[143, 144]	Mouse TAC and Ang II infusion	↓HDAC8 expression + Akt/Gsk3β signaling→ ↓ cardiac hypertrophy	[145]
miR-195-5p	↑ plasma	[146]	Mouse STZ-induced diabetic cardiomyopathy	↓ miR-195-5p → ↑ Bcl-2, ↑ sirtuin 1 → ↓ cardiac hypertrophy, ↓ ROS, ↓ apoptosis, ↑ myocardial capillary density, ↑coronary blood flow	[147]
			Rat cardiomyocytes	↓ miR-195-5p → ↑ SGK1 → rescues hERG potassium ion channel deficiency	[148]
miR-29a	↓ serum	[149]	Zucker diabetic fatty rats	↓ miR-29a → ↓ mcl-1	[150]
			Rat myocardial cells stimulated with high glucose	↓ IGF-1 → ↑ apoptosis	[151]
miR-222	↓ serum	[149]	Mice with diabetic cardiomyopathy	↓ Wnt/ β-catenin signaling → ↓ EndoMT → ↓ cardiac fibrosis, ↑ cardiac function	[152]
			IRI in cardiomyocyte-specific miR-222 OE mice	↓ p27/HIPK1/Hmbox-1 → ↑ growth/proliferation, ↓ apoptosis	[152]
			miR-222 OE in mice	↓ p27 → ↑ mTOR signaling, ↓ autophagy → ↑ hypertrophy, ↑ fibrosis, ↑ dysfunction with age	[153]

*Akt/Gsk3β* protein kinase B/glycogen synthase kinase 3 beta, *Ang II* angiotensin II, *bak1* Bcl2 homologous antagonist/killer*, Bcl-2* B cell lymphoma 2, *CD36* cluster of differentiation 36, *EndoMT* endothelial to mesenchymal transition, *HDAC8* histone deacetylase 8, *hERG* human Ether-a-go-go-Related Gene, *HIPK1* homeodomain interacting protein kinase 1, *Hmbox-1* homeobox containing 1, *I/R* ischemia/reperfusion, *IGF-1* insulin-like growth factor 1, *IRI* ischemia/reperfusion injury, *klf13* kruppel-like factor 13, *LAD* left anterior descending, *Mcl-1* myeloid cell leukemia 1, *MI* myocardial infarction, *miR* microRNA, *mTOR* mammalian target of rapamycin, *OE* overexpression, *ROS* reactive oxygen species, *SGK1* serum/glucocorticoid regulated kinase 1, *STZ* streptozotocin, *TAC* transverse aortic constriction, *VDAC1* voltage-dependent anion-selective channel 1, *Wnt* wingless-related integration site

Serum miRNAs are expressed differentially per trimester between healthy pregnant and GDM patients [138]. In the first trimester, miR-125b-5p expression is shown to be elevated in the serum of GDM patients compared to healthy pregnant women and normalizes in the second and third trimesters [138]. Patients with acute myocardial infarction (MI) are reported to have elevated serum miR-125b-5p levels compared to controls [154]. However, in the mouse heart, miR-125b-5p was shown to play a role in protection against MI by repressing pro-apoptotic genes bak1 andLC3 (klf13) in cardiomyocytes [139]. Two cardio-protective miRs, namely, miR-183-5p and miR-200b-3p, are shown to be elevated in GDM serum compared with healthy pregnant control serum in the first trimester of pregnancy but become significantly downregulated by the third trimester [138]. Indeed, in the male rat heart, miR-183-5p was shown to protect against MI by repressing mitochondrial voltage-dependent anion channel 1 (VDAC1) leading to decreased apoptosis upon ischemia/reperfusion injury [140]. Interestingly, miR-200b-3p has been shown to protect against cardiac fibrosis and cardiac dysfunction in STZ-induced diabetic cardiomyopathy by inhibiting cardiomyocyte apoptosis via pro-fibrotic CD36 repression [141] and by inhibiting endothelial-to-mesenchymal transition [142].

Several studies have shown that plasma levels of miR-21-3p and miR-195-5p are upregulated in GDM patients compared to controls [143, 144, 146]. While not much is yet known about the role of miR-21-3p in diabetic cardiomyopathy thus far, miR-21-3p is shown to play a role in cardiac hypertrophy and HF. MiR-21-3p protects against cardiac hypertrophy in male mice by regulating histone deacetylase 8 (HDAC8) expression and Akt/Gsk3β signaling, important for growth control in the cardiovascular system [145]. It has been shown that miR-195-5p expression is upregulated in the hearts of STZ-induced diabetic cardiomyopathy in male mice [147]. Here, silencing of miR-195-5p in STZ mice led to enhanced expression of pro-survival mediators B cell lymphoma 2 (BCL-2) and sirtuin 1. Furthermore, cardiac hypertrophy, ROS, and apoptosis as measured by caspase 3 activity were reduced upon miR-195-5p silencing in male STZ mice, while myocardial capillary density and coronary blood flow were improved [147]. Similarly, miR-195-5p expression in rat cardiomyocytes is upregulated by high glucose stimulation [148]. Here, it was shown that silencing miR-195-5p rescues high-glucose-induced hERG potassium ion channel deficiency by restoring serum and glucocorticoid-regulated kinase 1 (SGK1) expression [148].

Expression levels of miR-29a and miR-222 are reported to be significantly reduced in the serum of GDM patients compared to healthy pregnant controls in similar gestational weeks [149]. The miR-29 family consists of 3 members; miR-29a, -b, and -c, and is well-known to be involved in diabetes mellitus and diabetic cardiomyopathy pathophysiology [155]. Interestingly, however, miR-29 levels are usually elevated in serum and several tissues of diabetic patients and animal models [155]. Cardiac miR-29a expression is elevated in male Zucker diabetic fatty (ZDF) rats compared to male Zucker lean rats and is concomitant with reduced expression of anti-apoptotic myeloid cell leukemia-1 (mcl-1) gene expression [150]. Furthermore, miR-29a has been shown to promote apoptosis in rat myocardial cells stimulated with high glucose by repressing insulin-like growth factor 1 (IGF-1) [151]. How these reports relate to reduced serum miR-29a levels in GDM patients remains to be elucidated. Reduced expression of miR-222 is shown in both patients and experimental models of adverse cardiac remodeling and HF [156, 157]. In male mice with diabetic cardiomyopathy, miR-222 has been shown to diminish cardiac fibrosis and improve cardiac function [156]. Here, miR-222 mainly acts by inhibiting endothelial-to-mesenchymal transition in the myocardial microvasculature by suppressing Wnt/β-catenin signaling. Furthermore, male mice with inducible cardiomyocyte-specific miR-222 overexpression are shown to be protected against ischemia-reperfusion injury by preserving cardiac structure and function while decreasing scar formation [152]. Here, miR-222 inhibits apoptosis by directly targeting cyclin-dependent kinase inhibitor p27, homeodomain interacting protein kinase 1 (HIPK1), and Hmbox-1 in cardiomyocytes [152]. In contrast, it has been reported that male mice overexpressing miR-222 in a cardiomyocyte-specific manner develop cardiac hypertrophy, fibrosis, and dysfunction with age [153]. Here, miR-222 represses p27, leading to activation of mTOR signaling and subsequent inhibition of autophagy.

### Dysregulated miRNAs in preeclampsia and gestational hypertension

Numerous studies have shown differential expression of circulating miRNAs in pregnant females with PE, as has been reviewed previously [158, 159]. Here, we will focus on some of the prominent miRNAs that are known to play a role in adverse cardiac remodeling and HF (Fig. 1 and Table 2).

**Table 2:** Differentially expressed circulating miRNAs in preeclampsia and their effects in heart disease

miRNA	Regulation in human pregnancy	Ref.	Heart disease	Mechanism and outcome	Ref.
miR-210-3p and miR-210-5p	↑ serum	[160–163]	Hypoxic cardiomyocytes, mouse and rat	↑ Akt → ↑ miR-210 → ↓ PDCD4 → ↓ ROS, ↓ cell death	[164, 165]
			Mouse LAD ligation	↑ miR-210-3p → ↓ APC → ↓ cardiomyocyte apoptosis, ↑ angiogenesis, ↑cardiac function	[166]
			Mouse LAD ligation	↑ miR-210-3p in mesenchymal stem cells-derived extracellular vesicles → ↓ Efna3 → promote cardiac angiogenesis post-MI	[167]
miR-29a	↑ plasma	[168]	Patients with hypertrophic cardiomyopathy	↑ miR-29a → ↑ cardiac hypertrophy, ↑fibrosis	[169, 170]
			Mouse TAC	↓ miR-29a → ↓ cardiac hypertrophy, ↓fibrosis	[170]
			ET-1 in H9c2 cardiomyocytes	↓ NFATc4 → ↓ cardiomyocyte hypertrophy	[171]
miR-21	↑ plasma	[160]	Mouse, cardiac fibroblast-derived exosomes	↑ crosstalk between cardiac fibroblasts and cardiomyocytes → ↑ cardiomyocyte hypertrophy	[172]
			Mouse TAC	↓ *Spry1,* ↑ ERK-MAPK activity → ↑ fibroblast survival, ↑ growth factor secretion → ↓ fibrosis, ↓ cardiac hypertrophy	[172]
			Mouse post-MI via LAD ligation	↓ SMAD7 → ↑ cardiac fibrosis post-MI	[173]
			Rat IRI	↓ PDCD4 → ↓ apoptosis post-MI	[174]
			Mouse LAD ligation	↓ miR-21 → targets KBTBD7 (p38 MAPK and NFκB modulator) → ↓ cardiac dysfunction/inflammatory signaling	[175]
miR-155	↑ plasma	[160]	miR-155-KO mice TAC	↓ miR-155 → ↑ jumonji/Jarid2 → ↓ cardiac hypertrophy	[176]
			miR-155 KO macrophages	↑ Socs1 → ↑ cardiomyocyte hypertrophy	[177]
			fibroblast miR-155-KO	↓ TP53INP1→ ↑ cardiac remodeling	[178]
miR-206	↑ plasma	[179]	Mouse with TAC-induced cardiac hypertrophy	↑ miR-206 → ↓ tumor suppressor FoxP1→ ↑ TAC-induced cardiac hypertrophy	[180]
miR-144	↓ plasma and ↓ serum	[160, 161, 168]	miR-144-KO mouse LAD ligation	↑ Zeb-1→ impaired fibrotic response post-injury → cardiac dysfunction	[181]
			Mouse LAD ligation	↑ miR-144 mimic injection → ↓ fibrosis, ↓inflammation, ↓apoptosis → ↑cardiac function	[182]
			miR-144-KO mouse	↓ Rac-1 → ↑ ROS	[183]
miR-125b-5p	↓ plasma	[184]	Mouse LAD ligation	↓ bak1, ↓ klf13 → ↓ cardiomyocyte apoptosis	[139]
miR-195-5p	↓ plasma	[184, 185]	Severe PE patients	Unknown mechanism	NA
	↑ plasma	[186]	PE patients	↑ sFLT1 levels	[186]
			Mouse Ang II infusion	↑ miR-195-5p → ↓ FBXW7/MFN2 → ↑ mitochondrial membrane depolarization/ROS production → ↑ cardiomyocyte hypertrophy	[187]
miR-126	↓ serum	[161]	miR-126-KO mice LCA ligation	↑ Spred1 → defective angiogenesis	[188]
			Human cardiac microvascular endothelial cells	↑ miR-126 → ↑ PI3K/Akt, ↑ VEGF, ↑SOD expression → hypoxia/reoxygenation injury protection	[189]
miR-204	↓ serum	[161]	Mouse LAD ligation	↑ miR-204 → ↓ LC3-II	[190]
miR-15b	↓ serum	[161]	Mouse TAC overload	↑ miR-15b → ↓TGFβ signal(p38 MAPK/TGFβR-1 → ↑ cardiomyocyte hypertrophy, ↑ fibrosis	[191]

*Akt* protein kinase B, *Ang II* angiotensin II, *APC* adenomatous polyposis coli, *ECM* extracellular matrix, *Efna3* angiogenesis modulator ephrin A3, *ERK-MAPK* extracellular signal-regulated kinases/mitogen-activated protein kinase, *ET-1* endothelin 1, *FBXW7* F-box and WD repeat domain containing 7, *FoxP1* Forkhead box protein P1, *IRI* ischemia/reperfusion injury, *Jarid2* jumonji, *AT* rich interactive domain 2, *KBTBD7* kelch repeat and BTB domain-containing protein 7, *KO* knockout, *LAD* left anterior descending, *LC3-II* microtubule-associated protein 1 light chain 3, *LC3-II* microtubule-associated protein 1 light chain 3, *LCA* left coronary artery, *MFN2* mitofusion 2, *MI* myocardial infarction, *miR* microRNA, *NFATc4* nuclear factor of activated T cells 4, *NFκB* nuclear factor kappa-light-chain-enhancer of activated B cells, *p38 MAPK* mitogen activated protein kinase p38, *PDCD4* programmed cell death protein 4, *PE* preeclampsia, *PI3K/Akt* phosphatidylinositol 3-kinase/protein kinase B, *RAC-1* Ras-related C3 botulinum toxin substrate 1, *ROS* reactive oxygen species, *sFLT1* fms-like tyrosine kinase 1, *SMAD7* small mothers against decapentaplegic 7, *Socs1* cytokine signaling 1, *SOD* superoxide dismutase, *Spry1* sprouty RTK signaling antagonist 1, *TAC* transverse aortic constriction, *TGFβR-1* transforming growth factor beta receptor I, *TP53INP1* tumor protein p-53-inducible nuclear protein 1, *VEGF* vascular endothelial growth factor, *Zeb-1* zinc finger E-box binding homeobox 1

#### Upregulated miRNAs in preeclampsia

Elevated circulating levels of both miR-210-3p and miR-210-5p have been found in PE patients in several studies [160–163]. MiR-210, a hypoxia-activated miRNA, is upregulated in the heart in pathological hypertrophy and HF [192]. Interestingly, however, miR-210 seems to be cardio-protective. In cardiomyocytes, Akt was shown to increase miR-210 expression leading to reduced ROS and cell death, most likely by targeting programmed cell death protein 4 (PDCD4) mechanism [164, 165]. Additionally, miR-210 inhibits cell-cycle inhibitor adenomatous polyposis coli (APC), and miR-210-overexpressing female mice exhibited reduced cardiomyocyte apoptosis, upregulated angiogenesis, and overall improvement in cardiac function after MI [166]. A similar effect was observed in exosome-derived miR-210 that inhibits the angiogenesis modulator ephrin A3 (Efna3), thus promoting cardiac angiogenesis after MI in male mice [167].

In contrast to the downregulation in GDM, plasma miR-29a is upregulated in mild PE compared to healthy pregnant controls [168]. The miR-29 family plays dual roles in cardiac remodeling and HF [155]. In patients with hypertrophic cardiomyopathy, plasma miR-29a was found to be upregulated and to positively correlate with both cardiac hypertrophy and fibrosis [169, 170]. In TAC-induced cardiac pressure overload in male mice, inhibition of miR-29a attenuated cardiac hypertrophy and fibrosis [170]. However, miR-29a has also been shown to protect against phenylephrine-induced cardiomyocyte hypertrophy by directly targeting the pro-hypertrophic NFATc4 [171].

Circulating levels of specific miRNAs in PE may be different based on disease severity. miR-21 and -155 have been shown to be elevated in the plasma of PE patients, upregulated approximately 5–8-fold in severe PE compared to mild PE [160]. While its role remains controversial, miR-21 is thought to be one of the most dysregulated and abundantly expressed miRNAs in hypertrophic and failing hearts [193]. Increased miR-21 expression has been shown to induce cardiomyocyte hypertrophy by mediating crosstalk between cardiac fibroblasts and cardiomyocytes. MiR-21 inhibits sprout homolog 1 (Spry1) in cardiac fibroblasts, enhancing ERK MAPK signaling, leading to enhanced cardiac fibrosis and cardiomyocyte hypertrophy upon TAC-induced cardiac pressure overload in male mice [172]. MiR-21 also promotes cardiac fibrosis after MI in male mice by directly targeting small mothers against decapentaplegic 7 (SMAD7), a negative regulator of the TGF-β1 signaling [173]. However, cardio-protective effects of miR-21 are also reported. In a male rat model of cardiac ischemia/reperfusion, miRNA-21 protected against cardiomyocyte apoptosis by targeting PDCD4 [174]. In male mice, miR-21 attenuated cardiac dysfunction and inflammatory signaling after MI by directly targeting kelch repeat and BTB (POZ) domain containing 7 (KBTBD7), a modulator of p38 MAPK and NFκB signaling [175]. MiR-155 is a key mediator of cardiac inflammation and hypertrophy. MiR-155-deficient mice exhibited dampened cardiac hypertrophy upon TAC-induced pressure overload, most likely by relieving miR-155-induced inhibition of histone demethylase jumonji, AT rich interactive domain 2 (Jarid2) [176]. Loss of miR-155 in macrophages was shown to promote cardiomyocyte hypertrophy in a paracrine manner in male mice [177]. Here, miR-155 directly targets pro-hypertrophic suppressor of cytokine signaling 1 (Socs1). Additionally, miR-155 deficiency in male fibroblasts improved cardiac function and remodeling after MI through targeting tumor protein p-53-inducible nuclear protein 1 (TP53INP1) gene [178].

Interestingly, differences in circulating miRNA expression already before the onset of clinical symptoms may be predictive of PE development. Plasma miR-206 was upregulated in asymptomatic patients in the early third trimester who later developed PE compared to those who had a healthy pregnancy [179]. In male mice, miR-206 was shown to exacerbate TAC-induced cardiac hypertrophy by targeting tumor suppressor, Forkhead box protein P1 (FoxP1) [180]. Whether circulating miR-206 remains differentially expressed at the time of clinical PE manifestation remains to be elucidated.

#### Downregulated miRNAs in preeclampsia

Multiple studies have found plasma and serum miR-144 levels to be downregulated in PE patients compared to healthy controls, in various stages of disease progression [160, 161, 168]. Loss of miR-144 in male mice was shown to lead to impaired extracellular matrix remodeling after MI, leading to cardiac dysfunction. Here, miR-144 targets zinc finger E-box binding homeobox 1 (Zeb-1), a mediator of mesenchymal transition important for a proper fibrotic response after injury [181]. Conversely, injection of miR-144 mimics improved cardiac function after MI in mice by reducing fibrosis, inflammation, and apoptosis [182]. Additionally, loss of miR-144 in male mice enhances injury after MI by targeting Ras-related C3 botulinum toxin substrate 1 (Rac-1), a key component of NADPH oxidase, which results in elevated ROS levels [183].

In contrast to observed upregulation in GDM, plasma miR-125b-5p and miR-195-5p are shown to be downregulated in severe PE compared to healthy controls [184, 185]. However, elevated plasma miR-195-5p has also been reported in PE patients, where it positively correlates with sFLT1 levels [186]. In male mice, miR-195-5p promotes Angiotensin II-induced cardiomyocyte hypertrophy by targeting its downstream targets, tumor suppressor FBXW7, and mitofusin 2 (MFN2), which are known to inhibit mitochondrial membrane depolarization and ROS production [187].

Strikingly, differences in circulating miRNA expression levels before clinical PE symptoms are apparent may be predictive of future disease. Serum levels of miR-126, miR-204, and miR-15b in early gestation were found to be downregulated in women who developed severe PE in the third trimester, compared to women who developed a healthy pregnancy [161]. Endothelial cell and vascular integrity are regulated by miR-126. It was demonstrated that miR-126 represses the anti-angiogenic modulator sprouty-related, EVH1 domain-containing protein 1 (Spred1), leading to defective angiogenesis after MI in miR-126-deficient mice [188]. Furthermore, miR-126 protects human cardiac microvascular endothelial cells against hypoxia/reoxygenation injury by activating PI3K/Akt signaling and increasing VEGF and SOD expression [189]. MiR-204 seems to play a role in autophagy modulation. It was demonstrated that miR-204 may target cardiomyocyte microtubule-associated protein 1 light chain 3 (LC3-II), which is important for autophagosome formation, in cardiac ischemia/reperfusion injury in rats [190]. Lastly, miR-15b was demonstrated to inhibit several components of the TGFβ signaling pathway in cardiomyocytes including p38 MAPK and TGFβ receptor 1 (TGFβR-1), with in vivo miR-15b antagonism leading to enhanced cardiomyocyte hypertrophy and fibrosis upon TAC-induced pressure overload in mice [191].

#### Dysregulated miRNAs in gestational hypertension

GH and PE are related but distinct disorders, which is reflected in the circulating miRNA profile of PE and GH patients (Fig. 1 and Table 3). For instance, serum levels of miR-29a were shown to be increased in both PE patients and GH patients compared with normotensive patients [194]. Furthermore, plasma miR-125b-5p was downregulated in both PE and GH patients [184]. Interestingly, however, serum miR-181a was shown to be elevated in GH patients compared to normotensive and PE patients, in whom no difference in serum miR-181a levels was found [194]. It has been reported that miR-181a plays several roles in HF. Elevated plasma miR-181a has been suggested to be a marker of acute MI, where miR-181a levels positively correlate with the oxidative stress marker lipid hydroperoxide [195]. In a male rat model of MI, cardiac miR-181a expression increases over time and was shown to be associated with enhanced expression of the extracellular matrix components collagen I and fibronectin by directly targeting the anti-fibrotic TGF-β type III receptor in cardiac fibroblasts [196]. However, in a rat model of pressure overload cardiac hypertrophy via abdominal aortic constriction, cardiac miR-181a was reported to be downregulated. Downregulation of miR-181a in cardiomyocytes led to enhanced hypertrophy due to enhanced autophagy and expression of miR-181a target autophagy-mediated protein 5 (ATG5) [197].

**Table 3:** Differentially expressed circulating miRNAs in gestational hypertension and their effects in heart disease

miRNA	Regulation in human pregnancy	Ref.	Heart disease	Mechanism and outcome	Ref.
miR-29a	↑ serum	[194]	Patients with hypertrophic cardiomyopathy	↑ miR-29a → ↑ cardiac hypertrophy, ↑fibrosis	[169, 170]
			Mouse TAC	↓ miR-29a → ↓ cardiac hypertrophy, ↓fibrosis	[170]
			ET-1 in H9c2 cardiomyocytes	↓ NFATc4 → ↓ cardiomyocyte hypertrophy	[171]
miR-125-5p	↓ plasma	[184]	Mouse LAD ligation	↓ bak1, ↓ klf13 → ↓ cardiomyocyte apoptosis	[139]
miR-181a	↑ serum	[194]	Human with AMI or unstable angina	↑ oxidative stress marker lipid hydroperoxide	[195]
			Rat LAD ligation	↓ TGF-β type III receptor in cardiac fibroblasts → ↑ collagen I, ↑ fibronectin	[196]
			Rat abdominal aortic constriction	↓ miR-181a → ↑ ATG5 → ↑ hypertrophy	[197]

*AMI* acute myocardial infarction, *ATG5* autophagy-related 5, *GH* gestational hypertension, *LAD* left anterior descending, *MI* myocardial infarction, *miR* microRNA, *TGF-β* transforming growth factor-beta

### Dysregulated miRNAs in peripartum cardiomyopathy

While not many differentially-expressed circulating miRNAs have been identified in PPCM, the miRNAs that are known have directly been shown to contribute to PPCM cardiac pathophysiology (Fig. 1 and Table 4).

**Table 4:** Differentially expressed miRNAs in peripartum cardiomyopathy

miRNA	Regulation in human pregnancy	Ref.	Heart disease	Mechanism and outcome	Ref.
miR-146a	↑ plasma	[62, 198]	Cardiomyocyte-restricted STAT3-KO mice	↑miR-146a → ↓NRAS → ↓ EC proliferation, ↑ apoptosis	[62]
				↑ miR-146a in cardiomyocytes → ↓ ERBB4 → ↓ metabolic activity	[62]
miR-199a	↑ LV tissue	[61, 199]	Cardiomyocyte-restricted STAT3-KO mice	↑miR-199a-5p → ↓ERBB4 in cardiomyocytes → ↓glucose uptake, ↑ROS, ↑cell death	[199]
				↑miR-199a-5p → ↓ Ube2g1/Ube2i → cardiomyocyte sarcomere disarray ↑miR-199a-5p → ↑ ADMA secretion from cardiomyocytes → ↓ NO bioavailability, ↑ cardiac EC dysfunction, ↑ apoptosis	[61]

*ADMA* asymmetric dimethylarginie, *EC* endothelial cell, *ERBB4 Erb-B2 receptor tyrosine kinase 4, KO* knockout, *LV* left ventricle, *miR* microRNA, *NO* nitric oxide, *NRAS NRAS* proto-oncogene*, ROS* reactive oxygen species, *STAT3* signal transducer and activator of transcription 3, *Ube2g1* ubiquitin-conjugating enzyme E2 G1, *Ube2i* ubiquitin-conjugating enzyme E2 I, *UPS* ubiquitin-proteasome system

In plasma, serum, and myocardium of PPCM patients, miR-146a is well-known to be elevated [62, 198]. PPCM-associated anti-angiogenic 16kDa-PRL induces miR-146a expression via NFκB in endothelial cells [62]. It has been shown that miR-146a inhibits proliferation and enhances apoptosis of endothelial cells by repressing the proto-oncogene neuroblastoma RAS viral oncogene homolog (NRAS) [62]. Additionally, miR-146a is packed into endothelial cell-derived exosomes which can be taken up by cardiomyocytes [62]. In cardiomyocytes, miR-146a dampens metabolic activity through inhibition of receptor tyrosine-protein kinase erbB-4 (ERBB4), an important modulator of physiological pregnancy-induced cardiac hypertrophy [62]. Indeed, in both the STAT3-deficient PPCM female mouse model and PPCM patients, miR-146a is upregulated while ERBB4 expression is decreased compared to healthy controls [62].

Besides miR-146a, in the LV of STAT3-deficient PPCM male mice and PPCM patients miR-199a-5p was found to be upregulated [61, 199]. Here, decreased STAT3 levels induced miR-199a-5p-mediated ERBB4 inhibition in cardiomyocytes, leading to reduced glucose uptake by the heart, ROS production and cell death [199]. Furthermore, decreased STAT3 levels in cardiomyocytes were shown to induce miR-199a-5p-mediated repression of the ubiquitin-proteasome system (UPS) by repressing ubiquitin-conjugating enzymes Ube2g1 and Ube2i [61]. This ultimately leads to cardiomyocyte sarcomere disarray. Additionally, miR-199a-5p-mediated UPS dysfunction leads to enhanced secretion of asymmetric dimethylarginine (ADMA) from cardiomyocytes. In turn, secreted ADMA lowers nitric oxide bioavailability for cardiac endothelial cells, leading to endothelial dysfunction and apoptosis [61].

## Future perspectives and concluding remarks

In this review, we summarize current knowledge on pregnancy-related cardiovascular complications that may lead to cardiac dysfunction during pregnancy in previously healthy women, emphasizing the possible role of miRNAs in the cardiac pathophysiology of these complications.

Since about 12% of pregnancy-related deaths in the USA have been attributed to HF, and since GDM, PE, GH, and PPCM have been associated with a short- and long-term risk of HF development and death, there is a necessity for novel diagnostic and prognostic markers and therapeutic targets [9–12]. Circulating miRNAs have been proposed to fulfill these needs in both cardiac dysfunction and pregnancy-related complications [159, 200]. While the mounting data on circulating miRNA expression in pregnancy complications is promising, some discrepancies exist between studies. Such discrepancies may be due to differences in isolation and profiling of miRNAs either from plasma or serum, population characteristics, gestational age, internal controls, or normalization methods [159, 200].

Connecting circulating miRNAs in pregnancy-related cardiovascular complications to adverse cardiac remodeling and dysfunction in pregnancy remains understudied and further research needs to be conducted. However, several hurdles must be overcome. Firstly, all but a few studies have not directly linked circulating miRNAs to cardiac pathology since human cardiac tissue samples from pregnant women are scarce. Therefore, animal models provide an attractive alternative to further study the mechanisms and therapeutics of cardiovascular complications and HF in pregnancy. Although rodent pregnancies differ vastly from human pregnancies and not all aspects of human pregnancy can be translated in rodents, both do have similar cardiovascular adaptations to pregnancy [201]. Secondly, the majority of mechanistic studies into the roles of miRNAs in cardiac dysfunction have been performed in male animals. A growing body of evidence points towards differences in miRNA regulation of cardiac remodeling and HF between males and females [202, 203], thus posing an extra translational hurdle into the role of miRNA in cardiac remodeling and HF in pregnant females. Thirdly, miRNAs have been shown to exert opposite effects on cardiomyocytes and cardiac fibroblasts, leading to varied disease outcomes [155, 193]. Therefore, it is important to delineate from which cell-types the altered circulating miRNAs in pregnancy complications originate and on which cardiac cell types their modulatory effects are the largest. Lastly, differences in circulating miRNA expression already before the onset of clinical symptoms have been reported in PE [161, 179]. Focusing on such early-response miRNAs will aid in developing true prognostic biomarkers for pregnancy-related heart disease.

### Perspectives and significance

While existing data from different heart disease models are promising, further investigation is needed to directly and causally link miRNAs to cardiac pathophysiology in cardiovascular complications of pregnancy, which will aid in improved diagnosis and development of novel therapies.

## Data Availability

Not applicable.

## Abbreviations

16kDa-PRL: 16-kDa N-terminal fragment of prolactin
3'UTR: 3′ untranslated region
ADMA: Asymmetric dimethylarginine
Akt: Protein kinase B
APC: Adenomatous polyposis coli
ATG5: Autophagy-mediated protein 5
BCL-2: BCcell lymphoma 2
CRP: C-reactive protein
EF: Ejection fraction
Efna3: Ephrin A3
ERBB4: Receptor tyrosine-protein kinase erbB-4
ERK: Extracellular signal-regulated kinase
FoxP1: Forkhead box protein P1
GDM: Gestational diabetes mellitus
GH: Gestational hypertension
GLUT4: Glucose transporter 4
GSK3β: Glycogen synthase kinase 3β
HDAC8: Histone deacetylase 8
hERG: Human ether-a-go-go-related gene
HF: Heart failure
HIPK1: Homeodomain interacting protein kinase 1
IFN-γ: Interferon γ
IGF-1: Insulin-like growth factor 1
IL-6: Interleukin-6
Jarid2: Demethylase jumonji, AT rich interactive domain 2
JNK: c-Jun N-terminal
KBTBD7: Kelch repeat and BTB (POZ) domain containing 7
klf13: Kruppel-like factor 13
LC3-II: Microtubule-associated protein 1 light chain 3
LV: Left ventricle
MAPK: Mitogen-activated protein kinase
mcl-1: Myeloid cell leukemia-1
MEK1: Mitogen-activated protein kinase 1
MFN2: Mitofusion 2
MI: Myocardial infarction
miRNA: MicroRNA
mnSOD: Mitochondrial superoxide dismutase
mTOR: Mammalian target of rapamycin
NFAT: Nuclear factor of activated T-cells
NF-κB: Nuclear factor kappa-light-chain-enhancer of activated B cells
NRAS: Oncogene neuroblastoma RAS viral oncogene homolog
p706SK: Ribosomal S6 protein kinase
PAI-1: Plasminogen activator-1
PDCD4: Programmed cell death protein 4
PE: Preeclampsia
PGC-1α: Peroxisome proliferator-activated receptor gamma coactivator 1-alpha
PI3K: Phosphoinositide-3-kinase
PPCM: Peripartum cardiomyopathy
Rac-1: Ras-related C3 botulinum toxin substrate 1
ROS: Reactive oxygen species
sENG: Soluble endoglin
sFLT1: Soluble fms-like tyrosine kinase-1
SGK1: Serum and glucocorticoid-regulated kinase 1
SMAD7: Small mothers against decapentaplegic 7
SNS: Sympathetic nervous system
Socs1: Suppressor of cytokine signaling 1
Spred1: Sprouty-related, EVH1 domain-containing protein 1
Spry1: Sprout homolog 1
STAT3: Signal transducer and activator of transcription 3
STOX1: Storkhead box 1
STZ: Streptozotocin
TAC: Transverse aortic constriction
TGF-β1: Transforming growth factor-β1
TGFβR-I: Transforming growth factor-β receptor type I
TNF-α: Tumor necrosis factor α
TP53INP1: Tumor protein p-53-inducible nuclear protein 1
UPS: Ubiquitin-proteasome system
VDAC1: Voltage-dependent anion channel 1
VEGF: Vascular endothelial growth factor
WT: Wildtype
ZDF: Zucker diabetic fatty rat
Zeb-1: Zinc finger E-box binding homeobox 1
